# Acceptability of acceptance and commitment therapy for medication‐decision‐making and quality of life in women with breast cancer: A qualitative process evaluation

**DOI:** 10.1111/bjhp.12802

**Published:** 2025-05-13

**Authors:** Sophie M. C. Green, Louise H. Hall, Rachel Ellison, Jane Clark, Hollie Wilkes, Suzanne Hartley, Jay Naik, Sarah Buckley, Charlotte Hirst, Sue Hartup, Richard D. Neal, Galina Velikova, Amanda Farrin, Michelle Collinson, Christopher D. Graham, Samuel G. Smith

**Affiliations:** ^1^ Leeds Institute of Health Science University of Leeds Leeds UK; ^2^ Department of Health Sciences University of York York UK; ^3^ Department of Clinical and Health Psychology St James's University Hospital Leeds UK; ^4^ Clinical Trials Research Unit, Leeds Institute of Clinical Trials Research University of Leeds Leeds UK; ^5^ Department of Oncology Harrogate & District Foundation Trust Lancaster UK; ^6^ Department of Clinical Research Mid Yorkshire Hospitals NHS Trust Wakefield UK; ^7^ St James's University Hospital Leeds UK; ^8^ APEx (Exeter Collaboration for Academic Primary Care), Faculty of Health and Life Sciences University of Exeter Exeter UK; ^9^ Leeds Institute of Medical Research at St James's University of Leeds Leeds UK; ^10^ Leeds Teaching Hospitals NHS Trust Leeds UK; ^11^ Department of Psychological Sciences and Health University of Strathclyde Glasgow UK

**Keywords:** acceptability, acceptance and commitment therapy, adjuvant endocrine therapy, breast cancer, process evaluation, rapid assessment procedure

## Abstract

**Objectives:**

Adjuvant endocrine therapy (AET) reduces breast cancer recurrence, but side effects and distress impact adherence. We co‐designed an Acceptance and Commitment Therapy (ACT) intervention to support medication decision‐making and quality of life in women prescribed AET (ACTION). In a qualitative process evaluation nested in the pilot trial, we aimed to elicit participant experiences of receipt and therapists experience of delivery of ACTION to enhance our understanding of acceptability.

**Design:**

Remote semi‐structured interviews were conducted with women with breast cancer who received ACTION (*n* = 20) and trial therapists (*n* = 3).

**Methods:**

Interviews were guided by the Theoretical Framework of Acceptability (TFA). Rapid Assessment Procedure (RAP) sheets were completed after each interview to map responses onto TFA constructs, and sections of interviews were selectively transcribed. Individual RAP sheets were collated to identify key findings.

**Results:**

ACTION was generally liked, in particular, the group format (*affective attitude*). Participants and therapists felt ACTION was low effort, but therapists acknowledged the burden associated with trial procedures (*burden*). Participants generally felt able to engage with ACTION, and therapists felt they were able to deliver it (*self‐efficacy*). The perceived effectiveness of ACTION on well‐being was good, but was mixed for impact on treatment adherence (*perceived effectiveness*). Participants and therapists understood the aims of ACTION (*coherence*), and ACTION generally aligned with therapists' values (*ethicality*). Therapists questioned who would be most appropriate to deliver ACTION (*opportunity costs*).

**Conclusion:**

ACTION was acceptable to women with breast cancer and trial therapists. Rapid qualitative analysis can facilitate efficient process evaluations in time‐ and resource‐limited contexts.

**Statement of contributions**

**What is already known on this subject?**

- Adjuvant endocrine therapy (AET) can reduce the risk of recurrence of breast cancer, but adherence is low and is impacted by a range of factors.
- Acceptance and commitment therapy (ACT) could improve quality of life and treatment decision‐making in women with breast cancer prescribed AET, but higher quality research is needed.

**What does this study add?**

- ACTION, based on ACT, is acceptable for women with breast cancer and trial therapists.
- Demonstrates how process evaluations in resource‐limited contexts can benefit from rapid qualitative methods.

## INTRODUCTION

Most women with early‐stage breast cancer are prescribed adjuvant endocrine therapy (AET, e.g., tamoxifen, aromatase inhibitors) to reduce the risk of cancer recurrence and mortality (Early Breast Cancer Trialists Collaborative Group, 2011, 2015). However, up to three quarters of women miss doses or stop taking AET prematurely (Hershman et al., 2010; Huiart et al., 2013; Murphy et al., 2012; Partridge et al., 2003). Low adherence to AET increases the risk of recurrence and mortality, reduces quality adjusted life years and increases health care costs (Inotai et al., 2021; Makubate et al., 2013; McCowan et al., 2013).

A range of barriers to AET adherence have been reported, including the experience of unpleasant side effects (e.g., hot flushes, joint pain), psychological distress and unfavourable beliefs about AET (Cella & Fallowfield, 2008; Green et al., 2022; Jacobs et al., 2020; Lorizio et al., 2012; Toivonen et al., 2020). The most recent meta‐analysis of interventions aiming to support AET adherence included 25 unique studies and found an overall significant effect on adherence (Bright et al., 2023). However, the authors acknowledged a reliance on educational interventions alone which tend to be ineffective, and identified few interventions targeting psychological distress, depression, anxiety and negative affective attitudes towards AET (Bright et al., 2023). Following hospital‐based treatment, women with breast cancer face a variety of challenges that may contribute to psychological distress, including feeling abandoned due to the reduced level of professional support, fears and uncertainty surrounding recurrence, feelings of survivor guilt, processing their traumatic experience(s), difficulty coping with side effects and returning to ‘normal’ (Clancy et al., 2020; Hall et al., 2021; Jacobs et al., 2020; Moon, Moss‐Morris, Hunter, & Hughes, 2017; Peddie et al., 2021). Targeting a broader range of AET barriers could improve AET decision‐making, improve quality of life and support adherence to AET.

To support AET decision‐making and quality of life, we co‐developed, with women with breast cancer and healthcare professionals, an Acceptance and Commitment Therapy (ACT)‐based Intervention (ACTION) (Hall et al., 2021). The ACTION intervention aimed to enhance psychological flexibility, which is a process that involves approaching experiences with openness and awareness, and engaging in activities in line with one's values and goals (Hayes et al., 2006). Evidence suggests ACT is effective in reducing psychological distress and improving quality of life in people with physical health conditions, including cancer (Graham et al., 2016; Li et al., 2021). ACT skills such as eliciting values, mindfulness and unhooking (detaching from unhelpful thoughts and feelings) could improve quality of life and reduce distress by improving individuals' ability to live well alongside the emotional experiences that occur following a breast cancer diagnosis. ACT could also support women in making decisions around taking AET (Graham et al., 2021). For example, establishing whether AET aligns with their values, and using ACT‐based skills to cope with difficult emotions and side effects that may result from taking AET. There is some promising evidence that ACT may support treatment adherence; however, higher quality evidence is needed (Graham et al., 2021).

The ACTION pilot randomized controlled trial (RCT) randomized women with breast cancer prescribed AET to either usual care (UC) or UC + ACTION (Smith et al., 2022). Feasibility was demonstrated, and a priori progression criteria were met for recruitment, follow‐up, quantitative acceptability, competence and fidelity (Graham et al., 2024). In exploratory proof of principle analyses, there were signals of effectiveness in favour of the UC + ACTION arm for medication adherence, QoL, health‐related QoL, psychological distress and psychological flexibility (Graham et al., 2024).

We embedded a qualitative process evaluation in the ACTION pilot trial to assess the acceptability of the ACTION intervention. Assessment of acceptability is recommended by the UK Medical Research Council guidance as it offers the opportunity to make changes to improve acceptability ahead of a definitive evaluation trial (Moore et al., 2015; Skivington et al., 2021). Assessing acceptability to both the intervention deliverer and recipients at the pilot trial stage is useful, as a more acceptable intervention could lead to greater engagement among participants and increased fidelity of delivery by the intervention deliverers (Sekhon et al., 2017). The objective of this qualitative process evaluation was to understand the experiences of receiving and delivering the ACTION intervention to understand the acceptability of the ACTION intervention for women with breast cancer and therapists who delivered ACTION.

## MATERIALS AND METHODS

### Design

This qualitative process evaluation was nested in the ACTION pilot trial (ISRCTN: 12027752) which was a multi‐site, exploratory, two‐arm, individually randomized external pilot trial that randomized participants (1:1) to receive usual care or ACTION plus usual care (Graham et al., 2024; Smith et al., 2022). A qualitative process evaluation was included in the protocol.

For the process evaluation, semi‐structured interviews with trial participants and therapists were undertaken, guided by the Theoretical Framework of Acceptability (TFA) (Sekhon et al., 2017). The TFA was used as it conceptualizes acceptability as a multifaceted concept comprised of seven constructs: (1) affective attitude: how someone feels about an intervention; (2) burden: perceived amount of effort to participate or deliver the intervention; (3) self‐efficacy: confidence in performing behaviours to participate or deliver the intervention; (4) intervention coherence: understanding of the intervention and how it works; (5) perceived effectiveness: extent to which an intervention is perceived as likely to achieve its purpose; (6) ethicality: extent to which the intervention fits with individual values; (7) opportunity costs: extent to which anything must be given up to engage in, or deliver the intervention (Sekhon et al., 2017).

### ACTION intervention

ACTION was co‐designed with women with breast cancer and health care providers (Hall et al., 2021). It aimed to enhance psychological flexibility, which, in turn, was hypothesized to positively impact adherence to AET, quality of life and psychological distress (Graham et al., 2021). In accordance with stakeholder preference, ACTION comprised one 60‐min individual session with a therapist, three 90‐min group sessions, and access to an evidence‐based website with strategies for side effect self‐management and ACT exercises (Hall et al., 2022) (Appendix A). Due to the COVID‐19 pandemic, all sessions were remotely delivered using videoconferencing software.

The individual ACT session sought to improve engagement with later group sessions and the impact of later exercises by offering an initial personalized orientation to the psychological flexibility model and by initiating a therapeutic relationship with group facilitators. Conversations enabled exploration of some personal values and identification of important personal contexts (situations/moments/relationships). Tentatively, perspectives that begin to orient to psychological flexibility processes were offered to participants via observations of psychological experiences, options and actions. For example, possible workable/unworkable actions in the context of medication or side effects or, more broadly, in return to employment were explored. An overview of the ACTION intervention was provided. There were also home practice exercises designed to deepen connection with values.

The three group sessions involved experiential exercises designed to foster the six aspects of the psychological flexibility model (Hayes et al., 2006), within participants approach to everyday life at this stage of their breast cancer journey. The first session focused on expanding methods for approaching thoughts and feelings to include greater psychological flexibility. Participants were encouraged to track their psychological experiences, options and actions with granularity (present‐moment‐focus), noting workable and unworkable choices. Much of the content focused on defusion as a way to reduce barriers to workable actions. Participants were invited to experiment with common deliteralization tasks (e.g., ‘I am having the thought that’) or physicalizing exercises (thoughts as hands). The second session aimed to deepen connection with values and to encourage a wider set of committed actions (e.g. values compass, smallest possible step exercise). Here, a values‐based decision‐making framework was applied directly to medication management. Participants were invited to reflect on choices around taking/not taking medication and managing side effects from the perspective of their own values. Facilitators made participants aware of possible additional values‐congruent steps in the context of medication decisions (e.g., website side effect management suggestions, making an appointment with breast care nurse/oncologist). Willing responses to emotions/thoughts were more explicitly explored via the ‘passengers on the bus’ exercise. The home practice tasks were focused on encouraging and reinforcing new values‐consistent actions in everyday life. In the third session, via exercises like ‘notice who is noticing’ and metaphors/exercises involving approaching self‐stories as labels, participants were encouraged to notice possible benefits of taking a self‐as‐context perspective on their experiences. This session included an overarching reflection to identify and reinforce any effective behaviours learned across the whole intervention.

Participants were provided with an intervention booklet which contained the session plans, tasks and home‐practice tasks for in‐between sessions. Participants also had access to the ACTION website, which included strategies to manage AET side effects and the evidence base for each strategy, alongside supplementary ACT exercises (written information, audio clips and video clips) and videos of other women with breast cancer sharing their experiences, and signposting to further support (Graham et al., 2024). A full description of session content is provided elsewhere (Smith et al., 2022).

### Participants

Trial participants were adult women with stages I to IIIA breast cancer who had completed active hospital‐based treatment within the past 6 months and were prescribed AET (tamoxifen, raloxifene, anastrozole, letrozole and exemestane). Full trial eligibility criteria are described elsewhere (Graham et al., 2024). Participants were recruited via one of three routes: (1) a research nurse prospectively screened upcoming appointments to identify potentially eligible women; (2) women who self‐referred to see a healthcare professional due to AET side effects or adherence difficulties; and (3) retrospective screening of patient records. All participants randomized to receive ACTION plus usual care, who indicated willingness to be contacted about an interview, were sent an information sheet and consent form in the post after the final group ACT session of their cohort had taken place. We invited all interested participants with the aim to interview women from a range of ages, group cohorts and recruitment routes.

Trial therapists were Health and Care Professional Council (HCPC) registered practitioner psychologists (Clinical, Health or Counselling Psychologist). They received training regarding breast cancer and AET, psychological flexibility/inflexibility and ACTION‐specific therapy methods from two registered clinical psychologists with expertise in ACT. All therapists who indicated willingness to be contacted about an interview were sent an information sheet and consent form after they had delivered their final ACTION session.

### Data collection and analysis

All interviews took place via telephone or Microsoft Teams and were recorded either using an encrypted Dictaphone or MS Teams recording software. Interviews took place between February and August 2022, and were conducted by one researcher (SG), who had no prior involvement in the ACTION trial. The interviewer (SG) was an applied health researcher with experience in delivering low‐intensity psychological therapy. Participant interviews focused on the experience of participating in ACTION and the experienced acceptability of the ACTION programme (Appendix B). Therapist interviews focused on training, views on the content of the intervention, acceptability of intervention delivery and implementation of ACTION (Appendix C). Interview guides were used flexibly, with changes to the ordering and follow‐up questions based on participants' responses.

We used rapid qualitative methods for data analysis, due to the limited resources available (Vindrola‐Padros et al., 2022). A deductive approach was taken to analysis, using the TFA as a guiding framework. The interviewer took notes during the interview and completed a Rapid Assessment Procedure (RAP) sheet immediately after each interview (Appendices D and E) (Vindrola‐Padros et al., 2021, 2022). The RAP sheet was a two‐column table, with key topics guided by the interview guide and TFA in the first column, and space to input notes in the second column. An individual RAP sheet was completed for each participant and therapist to retain individual level data. Two overall RAP sheets collated findings for participants and therapists, respectively. Members of the research team (SG, SS, CG, LH and HW) attended regular meetings, approximately once a month, to discuss key findings, priority areas for future interviews and the need for further data collection. Participant and therapist quotes were selectively transcribed by one author (SG). To reduce bias, key findings were identified from the RAP sheets during team meetings, and the relevant section of the interview was then transcribed.

## RESULTS

Seventy‐nine participants were randomized in the ACTION pilot trial; 39 to usual care and 40 to ACTION plus usual care. Thirty‐eight (95.0%) participants randomized to ACTION agreed to be approached for interview, of which 20 (52.6%) provided consent. The remaining 18 participants did not respond to the interview invitation. Participant interviews were conducted between 1 month 11 days and 9 months 22 days after completion of the final ACTION session and lasted between 7 and 68 min (median = 52.5 min). Three out of four trial therapists consented to be interviewed. Therapist interviews were conducted between 9 and 65 days after completion of the final ACTION session and lasted between 46 and 56 min.

The mean age of interviewed trial participants was 60.0 years (SD = 9.61). Most interviewed trial participants were White British (19/20, 95.0%), postmenopausal (14/20, 70.0%) and taking anastrozole (14/20, 70.0%) (Table 1). The sample of interviewed participants was reflective of the trial participants overall. Demographic data including age, sex, job title, grade, length of time in current role and qualifications were collected for trial therapists, but are not reported as the sample is too small to preserve anonymity. A summary of the key findings relating to each domain of the TFA is presented below.

**TABLE 1:** Participant characteristics.

	UC (*N* = 39), *n* (%)	UC + ACTION (*N* = 40), *n* (%)	Interview (*N* = 20), *n* (%)
Age			
Mean (SD)	60.2 (10.17)	58.5 (10.62)	60.0 (9.61)
Recruitment route			
1. Recently completed treatment	17 (43.6)	15 (37.5)	7 (35.0)
2. Medication problems	1 (2.6)	0 (.0)	0 (.0)
3. Retrospective screening	21 (53.8)	25 (62.5)	13 (65.0)
Site			
Harrogate District Hospital	7 (17.9)	6 (15.0)	4 (20.0)
Mid Yorkshire Hospitals NHS Trust	15 (38.5)	15 (37.5)	5 (25.0)
St. James's University Hospital, Leeds	8 (20.5)	12 (30.0)	8 (40.0)
York Hospital	9 (23.1)	7 (17.5)	3 (15.0)
Ethnicity^a^			
White British	38 (97.4)	38 (95.0)	19 (95.0)
Other	1 (2.6)	2 (5.0)	1 (5.0)
Marital status^a^			
Married	24 (61.5)	27 (67.5)	13 (65.0)
Other	15 (38.5)	13 (32.5)	7 (35.0)
Employment status^a^			
Full/part time	19 (48.7)	19 (47.5)	8 (40.0)
Retired	14 (35.9)	11 (27.5)	8 (40.0)
Other	6 (15.4)	8 (20.0)	3 (15.0)
Missing	0 (.0)	2 (5.0)	1 (5.0)
Education^a^			
Degree level or above	18 (46.2)	12 (30.0)	6 (30.0)
Higher educational qualifications	5 (12.8)	9 (22.5)	6 (30.0)
Other	16 (41.0)	16 (40.0)	7 (35.0)
Missing	0 (.0)	3 (7.5)	1 (5.0)
Cancer incidence at randomisation^a^			
First primary	37 (94.9)	37 (92.5)	19 (95.0)
Other	2 (5.1)	1 (2.5)	1 (5.0)
Missing	0 (.0)	2 (5.0)	0 (.0)
Current stage of cancer^a^			
Stage IA/IB	18 (46.2)	18 (45.0)	8 (40.0)
Stage IIA	15 (38.5)	16 (40.0)	9 (45.0)
Other	6 (15.4)	6 (15.0)	3 (15.0)
Menopausal status^a^			
Pre‐menopausal	5 (12.8)	6 (15.0)	2 (10.0)
Post‐menopausal	27 (69.2)	26 (65.0)	14 (70.0)
Other	7 (17.9)	8 (20.0)	4 (20.0)
Breast cancer treatment received^a^, ^b^			
Surgery: Lumpectomy	30 (76.9)	25 (62.5)	13 (65.0)
Surgery: Unilateral mastectomy	10 (25.6)	14 (35.0)	7 (35.0)
Surgery: Double Mastectomy	0 (.0)	0 (.0)	0 (.0)
Radiotherapy	19 (48.7)	22 (55.0)	10 (50.0)
Other treatment	6 (15.4)	12 (30.0)	4 (20.0)
Hormone therapy regimen^a^, ^b^			
Tamoxifen	15 (38.5)	12 (30.0)	6 (30.0)
Anastrozole	22 (56.4)	27 (67.5)	14 (70.0)
Other (Raloxifene, Exemestane and Letrozole)	2 (5.1)	3 (7.5)	1 (5.0)

Abbreviation: UC, usual care.

^a^ Data have been grouped to into other to preserve anonymity.

^b^ More than one response could be provided.

### Affective attitude

#### Individual ACT session

Nearly all participants reported liking the individual session at the beginning of ACTION:

It was quite nice to connect with the psychologist, and to talk about my particular situation…it was nice to just talk to somebody, to talk about my own personal journey (Participant 03, 51–69 years old)

A minority of participants had some reservations about the individual session:

I thought it left me a little bit, inquisitive to what was to come…my main question was at the end of this trial am I gonna be left with coping mechanisms to move forward (Participant 12, ≤50 years old)

I don't know whether I would have felt exactly the same had that session not have happened…I'm not sure I felt it was very necessary at that point (Participant 20, ≤50 years old)

Therapists had mixed opinions about the individual sessions, suggesting they liked them but would need more time for patients with more distress:

I thought the first few I did I found quite challenging, because often it was just a lot of information to get into the formulation…I think it was a nice way to build a rapport with somebody, to help answer their questions about the group, and just to give them a bit of a flavour I suppose about what they could expect….I think I was worried beforehand about it not being long enough….but actually it did work out quite well, I think because the people that we saw seemed to be managing pretty well too….I think probably if you had people who were struggling a bit more it would have felt like a lot to cover in the hour to be able to give them the proper time for them to talk about what was going on. (Therapist)

#### Content of group ACT sessions

Overall, participants were very positive about the content of the group‐based ACTION sessions, reflecting on several of the ACT skills that were taught:

The one about the bus, love the bus one, the analogy of the bus, I just thought that was amazing (Participant 14, ≤50 years old)

It reminds you of what's important, what you kind of lost focus on, and for me it was spending time with my family and my loved ones…not only because of COVID but because I didn't want to talk about what was happening to me. And I think that booklet made me realise that that's one of the most important things in my life and I've got to, you know, I've got to move forward, and continue spending time with the people that I care most about. (Participant 12, ≤50 years old)

A few participants did not find the content of the sessions useful:

I found them a bit obscure I think…I tend to just get on with things and I try not to dwell and yeah…I couldn't relate them to me, the, the exercises….more like a paper exercise than anything else (Participant 10, 51–69 years old)

I can't say that I found them really helpful…I think they weren't for me, it wasn't anything that I wouldn't have learnt about anyway…exercise and mindfulness and that sort of thing…I think its things that I think if you read magazines or, you know, articles in newspapers and things I think it's the sort of thing that you would know anyway. (Participant 17, ≥70 years old)

#### Format of group ACT sessions

Nearly all participants and all therapists acknowledged the benefits of ACTION being delivered in a group format:

Knowing that there's other ladies going through exactly the same…you don't sort of feel as isolated. (Participant 09, 51–69 years old)

I really think the group setting of the intervention was really helpful [for the participants], in having that peer support. (Therapist)

However, a small minority of participants felt uncomfortable in the group setting, due to comparisons of their own experiences with others:

Perhaps I thought, why aren't I panicking about this, why aren't I worrying about that, and it made me really quite anxious for a while afterwards. (Participant 10, 51–69 years old)

Reflecting on the benefits of meeting other women going through breast cancer, several participants suggested they would have liked more opportunity for social interaction:

I think we all said afterwards that it would have been nice to have a, just a get together later on you know just an hour, just to chat to see what was going with everybody and how everybody was doing. (Participant 09, 51–69 years old)

More free interactive time if it was going to be on Zoom, for those chats and what have you. (Participant 16, 51–69 years old)

#### Website

There were mixed opinions regarding the website component of ACTION. Some women reported elements of the website that they liked:

I liked a lot of the mindfulness things that were on there, I liked, just the relaxing things, you know helping you relax and things. (Participant 09, 51–69 years old)

I quite liked that they had little videos on of people talking, but I think they should've had more, I would have liked to have seen a lot more people's experiences, that really normalises it. (Participant 14, ≤50 years old)

However, some participants questioned the usefulness of the information, and the usability:

There are so many websites that you can go onto to do with breast cancer…I'm not sure that I couldn't have found the information elsewhere if I needed to, it was very simple, very clear. (Participant 08, 51–69 years old)

I'm not really great technically, so for me it was probably a bit clunky. (Participant 09, 51–69 years old)

### Burden

#### Participant burden

Participants generally reported it being easy to engage with ACTION:

I think it was easy to engage…what I did like was people running the zoom meeting, didn't say oh [name]… what do you think, because I wouldn't have liked that. (Participant 19, 51–69 years old)

The online format of the intervention was frequently spoken about in relation to burden, with mixed opinions. Some participants felt the online format was burdensome because it was more difficult to connect with the other participants, and most participants felt they would have preferred face‐to‐face sessions:

I think they would've been a lot better if we could've all been there in person…so I think it made it a little bit harder because you know when you're talking about such personal things you do sort of tend to try and form a little bit of a bond with people and I think that's much harder online than it would've been if we'd have all been sat around a table having a discussion, so I didn't really like that part of it. (Participant 13, 51–69 years old)

You got to the point where the screen was full, and there was another screen, you slide people across because there was so many people…I mean I know I've only got an iPad mini. (Participant 16, 51–69 years old)

However, a number of participants preferred the online format of ACTION, for both emotional and practical reasons:

I would have found it even more nerve wracking actually, having to go and meet them face‐to‐face in an unfamiliar place as well. (Participant 15, ≤50 years old)

Obviously you weren't travelling so that, you know, made it much easier to actually attend. (Participant 08, 51–69 years old)

#### Therapist burden

Therapists reported the burden of delivering ACTION was low. However, all therapists acknowledged the administration time associated with completing trial documents was time consuming:

There was a lot of, admin difficulties [laughs], and there was a lot of kind of process issues in setting things up, and sending out zoom links and filling out all the forms for the research file, but as far as the actual delivering the intervention part went, I think particularly for the group sessions it was really easy to deliver and it was very nicely structured and designed with a lot of detail, so you didn't have to do much preparation really. (Therapist)

### Self‐efficacy

#### Participants

Participants generally reported confidence in engaging with the ACT sessions, valuing the combination of booklets, home practice tasks and support from therapists to clarify understanding:

They did explain things afterwards, so if you felt that you'd perhaps not answered it correctly or not understood it, at least then you knew that was the way to think about it, or that was what was expected, you weren't sat there thinking oh I can't write anything because I can't think what to write. (Participant 07, ≥70 years old)

Self‐efficacy was lower for engagement with the website, with some women reflecting on technical difficulties of accessing the website:

People are saying you need to set up an account, well to me set up an account, you're saying you're going to be paying for something…it's modern language and I don't understand it always. (Participant 02, ≥70 years old)

#### Therapists

Two therapists felt confident delivering ACTION, due to the manualised nature, but one noted lower confidence for delivering the individual session:

Very [confident]…well I think because it's manualised isn't it, so it's very easy to adhere to. (Therapist)

I think I was personally a lot more worried about the individual sessions…I was very nervous about making sure I was being compliant to the model, and trying not to just fall into the, the clinical assessment that I would be doing within my clinical work, and, um, trying to make sure I got the whole formulation filled in and fed back within the time limit, I was a lot more nervous about my ability to do that. The group sessions I felt a lot more comfortable with. I think again because they were a lot more structured for us, and because we had a lot more of a detailed plan for what to do for them. (Therapist)

### Perceived effectiveness

Therapists and participants both reflected on the potential effectiveness of ACTION for improving quality of life and medication adherence, citing specific ACT skills:

My experience was, you know, it really made me think about why I had to take them [medication]… you are given your meds and you go away and there's not that much information and you're plunged into a horrible menopause and all of these things…for me it just made me think I've got to keep taking them…I imagine that would be useful, I'm sure I'm not the only person who's struggled with them… so any support regarding those meds is vital I think. (Participant 13, 51–69 years old)

I'm using it every day to stress, to manage stress… grounding, being aware, just sitting down, and writing down what's important to you like your family, your relationships, you know, like re‐honing on here and now… it's been a game changer for me. (Participant 04, 51–69 years old)

In terms of quality of life, I would imagine it's very helpful…um, because it's keeping them in touch with their values and what's important. (Therapist)

In addition, most participants felt that they would recommend ACTION to others in a similar situation:

Yeah definitely… because of what you get out of it….it changed my whole outlook on life, I'm a totally different person to what I was when I started the sessions. (Participant 19, 51–69 years old)

I would definitely recommend it, yeah, without a doubt…I think it helps process them thoughts, in moving forward, because if you don't you're just gonna be stuck in this vicious circle of worry and threat, and so I think having that opportunity, to, to speak to somebody in regards to how you do make them steps in moving forward…it is a benefit for a lot of people. (Participant 12, ≤50 years old)

There were more mixed opinions regarding how effective ACTION would be for supporting adherence to AET specifically:

It hasn't felt like it's been something, a programme that's, that would have had an impact on adherence…its felt like more something that would've been about improving quality of life, and I feel like that would probably be a better focus…I think ACT probably could [impact adherence], I think it just, well it's probably that adherence wasn't an issue for these women so it's hard to say I guess. (Therapist)

It felt like it works more for the wellbeing factor than the medication factor…there could be a little bit more information there about, about the medication, but as far as the wellbeing side of it, I think that, that worked absolutely fine. (Participant 12, ≤50 years old)

Therapists also noted that ACTION worked well for the trial participants who were generally coping well, but felt adaptations may be needed for those in more distress:

The women who seemed to benefit the most from it were women who probably were naturally quite reflective, were actually coping really well….if we had women in who had a lot of issues and concerns I think that would've made it more challenging…I think there probably wouldn't have been enough time because some of the exercises would've taken a bit longer potentially. (Therapist)

### Intervention coherence

Participants reported a general understanding of the aims of the ACTION programme, including reflections on the purpose of the skills and the benefits of the group setting in particular:

For me, I think it normalises cancer, it makes you feel that you're not on your own. It makes you understand, or helps you to understand some of the side‐effects, and because the facilitators are there, some of them could answer some of the questions and sort of like say, and give you tips and things and the website supports that as well. And it just gives you techniques on how to deal with negative thoughts, negative feelings, overwhelming feelings, and gives you just like an arsenal of techniques that you can put into place so that you can get on with your life and, and live your life as full. (Participant 14, ≤50 years old)

Therapists were clear on the aims of ACTION:

It covers certain skills, ACT skills, to help people think about how they relate to their thoughts and feelings, the kind of short term and long‐term consequences, or impact of that, and helps people then to make choices about what they may or may not want to do, and also helps people to think about what's important to them so that they're living a kind of meaningful life. (Therapist)

### Ethicality

Overall, all therapists felt ACTION fit with their values, but two therapists highlighted specific aspects of ACTION that aligned less with their professional values. One therapist noted some misalignment due to the focus on one psychological model:

It's aligned in some ways in terms of quality of life, you know obviously I would look at that in my values as a therapist, to improve people's quality of life, health and wellbeing, psychological resilience and ability to cope, so it's aligned in that sense. I guess it would be misaligned, or less aligned, are bits of how that's done, so again like with the bringing in different models… So, yeah fairly consistent with my values. (Therapist)

Another therapist felt the group sessions aligned with their values, but the individual session did not:

I think the group work really fit with my values as a therapist. I think I struggled quite a bit with the individual sessions, because I didn't feel like I was able to allow people the time to talk that they needed, and that I really struggled with, because it felt like we were opening that door, but also saying you can only talk about this for another 50 minutes. (Therapist)

### Opportunity costs

The only opportunity cost, mentioned by two participants, was the potential interference of ACTION upon returning to their work‐life routine:

I was having to think about things that I didn't want to think about because I'd actually got into a routine of going back to work, so everything was now normal. (Participant 18, 51–69 years old)

Therapists noted that they had to give up clinic time to deliver ACTION, and questioned what level of therapist would be most appropriate to deliver ACTION if it were to be implemented:

I guess time was a big thing from the clinical perspective. We definitely noticed the impact within our waiting list. (Therapist)

I think the challenge would just be thinking about who delivers it really…I think it probably doesn't need to be a clinical psychologist, but I think it probably does need to be someone with more of a therapeutic background than a clinical nurse specialist really. Or it could be a clinical nurse specialist in conjunction with a psychologist, and then maybe eventually they could run it by themselves. (Therapist)

## DISCUSSION

This qualitative process evaluation, nested in the ACTION pilot randomized trial, demonstrated overall acceptability of the ACTION intervention to women with breast cancer and therapists delivering the intervention. Our use of the TFA provided an in‐depth, multifaceted assessment of acceptability, and the rapid qualitative methods enabled completion of the study in a time‐ and resource‐limited context, allowing summaries of findings to be communicated quickly.

The findings from this process evaluation offered valuable insights into adaptations that could be made to improve the acceptability of ACTION. Potential adaptations for consideration include more time for social interaction or exchanging of contact details to facilitate social connections, improvements to the usability of the website, offering sessions outside of working hours and offering an option for face‐to‐face sessions. From an implementation perspective, key considerations prior to further evaluation include whether different levels of therapists or other health care practitioners would be appropriate to deliver ACTION, and whether ACTION would be appropriate for women with higher levels of distress who are more likely to be seen in routine clinical practice. These adaptations should be considered ahead of a phase III randomized controlled trial.

The group‐based format of ACTION was appraised positively by most participants and therapists. Interview data suggested that for many women, the feelings of normalization from meeting other women going through a similar experience was a core component of ACTION. It is possible this feeling was enhanced due to the timing of the intervention; many of the women taking part in ACTION had undergone breast cancer treatment during the COVID‐19 pandemic, with reduced opportunities for meeting other women also undergoing treatment or attending support groups. Our results highlight the perceived benefits of social support, which has previously been associated with improved adherence to AET and QoL (Lambert et al., 2017; Lin et al., 2017; Moon, Moss‐Morris, Hunter, Carlisle, & Hughes, 2017; Murphy et al., 2012; Zhang et al., 2017). Social support could be explored as a mediating variable, alongside psychological flexibility, in any further evaluation of the ACTION intervention.

The quantitative ACTION pilot trial results indicated a small improvement in the total medication adherence (Adherence Starts with Knowledge Questionnaire [ASK‐12]) score from baseline to 6 months for usual care + ACTION over usual care, in a non‐powered proof of principle analysis (Graham et al., 2024). However, in the interviews, there were mixed opinions regarding the perceived effectiveness of ACTION in supporting treatment decision‐making and adherence to AET specifically. To some extent, lower perceived effectiveness for nonadherence could be expected as the relationship between ACT and treatment adherence may not be immediately clear to participants. It is hypothesized that ACT skills will increase psychological flexibility (e.g., engagement, openness and awareness), which can support decision making more generally, including adherence behaviours (Graham et al., 2021). Furthermore, improved psychological flexibility can improve quality of life and mood which, as these factors are associated with adherence (Bright et al., 2023; Green et al., 2022; Toivonen et al., 2020), may also create a context where effective decision‐making is likely to occur. Providing additional explanation for participants as to how ACT could impact treatment decision‐making could be beneficial for ACT interventions aiming to support this behaviour, as higher perceived effectiveness may or may not increase acceptability and engagement with an intervention (Sekhon et al., 2017). It may be possible to target ACT methods much more focally on medication decision‐making or treatment adherence than in our intervention (Arch et al., 2022). This would likely make the logic of the proposed intervention clearer to participants, but there may be drawbacks to doing so. A very focal intervention may appeal to a subgroup of those who struggle to manage medication and want help for this specifically. However, we were interested in enhancing decision making in the broad population of those prescribed AET. Furthermore, a narrow focus may not impact the wider range of factors, including mood and quality of life, that affect medication decision making.

The TFA was a useful framework to guide our multifaceted assessment of acceptability, and led to a greater understanding of acceptability compared to if we had asked about acceptability more generally (Sekhon et al., 2017). However, our experience suggests that certain constructs may vary in applicability across participant groups. For example, when asking participants whether the ACTION intervention fit within their personal values (ethicality), several participants were unsure exactly how to respond. This was surprising, given the ACTION intervention included exercises related to identifying personal values. In contrast, when asking therapists how ACTION aligned with their professional values, responses were more forthcoming, which is expected given therapists trained in ACT will be familiar with values terminology and what the question is trying to elicit. More broadly, it is possible that ethicality may be most relevant to assessments involving healthcare professionals, whereby they can assess alignment of an intervention with their professional values. Further guidance and example questions relating to the ethicality construct for research participants would be beneficial.

The rapid qualitative methods used in this process evaluation enabled the study to be undertaken within limited resource constraints. The RAP sheets produced immediately after each interview enabled findings to be communicated quickly to stakeholders, and for any necessary adaptations to the intervention to be considered prior to a definitive phase III trial. The selective transcription reduced the cost but still required researcher time, and could have led to some researcher bias compared to using full transcription (Vindrola‐Padros & Johnson, 2020). Since the interviews were conducted, in‐built transcription in videoconferencing platforms (e.g., Microsoft Teams) has improved considerably, and is a helpful tool for rapid analysis. Relevant quotes from the transcript available immediately after the interview can be inputted into the RAP sheet in a third column, thus further speeding up the rapid analysis process while retaining the participant's language (Green et al., 2024). With these improvements to the methods, researchers should consider using rapid qualitative methods for process evaluations in time or resource‐limited contexts.

### Limitations

The participant interviews were conducted between 1 month 11 days and 9 months 22 days after participants had attended their last ACT session, and therapist interviews were conducted between 9 and 65 days after intervention delivery. Therefore, interviews may be susceptible to recall bias. Nineteen out of 20 interviewed participants had attended all four sessions in ACTION, which was not representative of all participants allocated to this intervention within the trial. Moreover, 18 participants invited to the interview did not respond to the invitation for the interview. These participants may have had different views on the acceptability of ACTION. The majority of interviewed participants were White British, which reflected participants in the overall trial. Acceptability of psychological and group‐based interventions may differ in underrepresented ethnic minority groups (Arundell et al., 2021), which should be explored in greater detail in any future evaluations of the ACTION intervention.

## CONCLUSION

Overall, we have demonstrated the acceptability of the ACTION intervention in women with breast cancer and trial therapists. We have described an efficient approach to conducting a rapid process evaluation while maintaining an in‐depth, multifaceted assessment of acceptability. Taken together with the results of the ACTION pilot trial, which demonstrated feasibility, a phase III trial to evaluate the ACTION intervention is warranted.

## AUTHOR CONTRIBUTIONS

**Sophie M. C. Green:** Conceptualization; methodology; formal analysis; investigation; resources; project administration; writing – original draft; writing – review and editing. **Louise H. Hall:** Methodology; formal analysis; investigation; resources; writing – review and editing. **Rachel Ellison:** Methodology; resources; project administration; writing – review and editing. **Jane Clark:** Methodology; funding acquisition; writing – review and editing. **Hollie Wilkes:** Methodology; formal analysis; resources; project administration; writing – review and editing. **Suzanne Hartley:** Methodology; writing – review and editing. **Jay Naik:** Methodology; writing – review and editing. **Sarah Buckley:** Methodology; writing – review and editing. **Charlotte Hirst:** Methodology; writing – review and editing. **Sue Hartup:** Methodology; writing – review and editing. **Richard D. Neal:** Methodology; funding acquisition; writing – review and editing. **Galina Velikova:** Methodology; funding acquisition; writing – review and editing. **Amanda Farrin:** Methodology; funding acquisition; writing – review and editing. **Michelle Collinson:** Methodology; formal analysis; resources; funding acquisition; writing – review and editing. **Christopher D. Graham:** Conceptualization; methodology; formal analysis; investigation; resources; supervision; funding acquisition; writing – review and editing. **Samuel G. Smith:** Conceptualization; methodology; formal analysis; investigation; resources; supervision; funding acquisition; writing – review and editing.

## FUNDING INFORMATION

The trial was supported by funding from Yorkshire Cancer Research (L417) (PIs: Smith and Graham). This report is independent research supported by the National Institute for Health Research NIHR Advanced Fellowship, Prof Samuel Smith NIHR300588. Smith also acknowledges funding support from a Yorkshire Cancer Research University Academic Fellowship. CDG is part funded via several grants from NIHR. MC reports NIHR, Yorkshire Cancer Research, Macmillan Cancer Support, Breast Cancer Now and British Lung Foundation grant funding paid to their institution. AJF reports NIHR grant funding paid to their institution. The views expressed in this publication are those of the author(s) and not necessarily those of the NHS, the National Institute for Health Research or the Department of Health and Social Care. The funders had no role in the design of the study, data collection, analysis, interpretation of data and in the writing of this manuscript.

## CONFLICT OF INTEREST STATEMENT

SS declares personal consultancy fees from Lilly. CDG declares consultancy fees via NIHR. JC declares receiving honoraria from Novartis. JN declares receiving indirect payment from AstraZeneca and direct payment from the Breast Cancer Research Foundation. MC declares being a DMEC and TSC member of NIHR funded projects and being a member of NIHR grant funding panels. AJF declares being a DMEC and TSC member of NIHR and British Heart Foundation funded projects and an NIHR Senior Investigator. All other authors declare no conflicts of interest.

## Data Availability

The terms of the participant's consent do not allow raw data to be made publicly available. The interview guides used are shared in the appendices.

## APPENDIX A The TIDieR (Template for Intervention Description and Replication) Checklist

Original source: Graham CD, Ellison R, Hall LH, et al. A Pilot Randomised Controlled Trial of Acceptance and Commitment Therapy for Medication Decision‐Making and Quality of Life in Women With Breast Cancer: The ACTION Trial. *Psychooncology*. 2024;e6349. https://doi.org/10.1002/pon.6349.

Information to include when describing an intervention and the location of the information.

**Table:**

No.	What	Details
1	Name	The Yorkshire Cancer Research ACTION Pilot Trial: An ACT‐based intervention for women with breast cancer to support wellbeing and hormone therapy medication decisions
2	Why: Rationale, theory, goal	Adjuvant hormone therapies are prescribed at the end of hospital‐based breast cancer treatment in order to prevent recurrences and all‐cause mortality. However, adherence to these medications is often poor, due in part to intolerable side effects. This time during the cancer journey is also particularly challenging psychologically, as women are transitioning from ‘patient’ to ‘survivor’. Women also report a lack of support during this time, post hospital discharge. Previous adherence interventions have focussed on information giving and/or problem‐solving difficulties with medication‐taking (e.g., disorganized regimens), and have had little effect. Given the wide range of additional emotional (e.g., mood, motivation) and somatic (e.g., side effects) factors that contribute to low adherence, it is perhaps unsurprising that these previous interventions have been unsuccessful. An alternative, and potentially more effective strategy, is to design an intervention to target a range of emotional and physical factors that may affect adherence. Furthermore, using co‐design methods may be particularly beneficial, to ensure that any proposed intervention is acceptable to patients, as well as feasible to implement within routine NHS care. Acceptance and Commitment Therapy (ACT), is potentially well suited to tackle this problem, and has been shown to improve outcomes in those living with chronic illness, chronic pain and cancer. ACT is particularly suited to helping people function effectively in objectively difficult situations (e.g., living with illness or immutable pain). ACT aims to increase a participant's awareness of their personal values, and to undertake more of the behaviours that support these values—a process that often involves developing a willingness to have painful thoughts and feelings (such as medication side effects). Given the above, we have co‐designed and conducted a small run‐through of an ACT intervention for women with breast cancer who have been prescribed adjuvant hormone therapies. The aim of the intervention is to support well‐being, and hormone therapy medication decisions and adherence. This pilot RCT aims to feasibility test this intervention
3	What Materials	Practitioner psychologists delivering the intervention received 2 days of bespoke training delivered by clinical psychologists with ACT and cancer expertise. Alongside this, they received a training manual, with information about ACT generally, and specific session plans for the intervention sessions. Participants received an intervention manual, containing the session plans, tasks and homework tasks for in‐between sessions. Participants also had access to a bespoke website, containing additional ACT exercises and information, strategies for self‐management of side effects and signposting to further sources of support
4	What Procedures	*Clinician Training* (See section 5) *Intervention delivery* 1× Individual session with a practitioner psychologist delivered remotely via a video platform. 3× Group sessions with a practitioner psychologist delivered remotely via a video platform. ACT tasks to practice at home between sessions. Website to access additional resources. *Evaluation of the Clinician Training* ACT Knowledge questionnaire administered post‐training. Clinician fidelity evaluated using the ACT‐FM completed by an external rater. Clinician fidelity to intervention procedures involved clinician self‐rating using a procedural fidelity checklist. Clinician competency was assessed by Dr. Graham, using the ACT‐FM, through review of audiotapes from the first individual session of all therapists at the treatment delivery site. *Evaluation of the Intervention* Adherence, Quality of Life/Symptom Burden, Anxiety and Depression measured pre‐ and post‐intervention. Acceptability of the intervention. Website use tracked. *Support activities* Recruitment and consent of participants
5	Who provided	The practitioner psychologists delivering the intervention underwent training in delivering Acceptance and Commitment Therapy. The training was delivered by Dr. Chris Graham (CG), who has expertise in ACT applied to chronic disease, and Dr. Jane Clark (JC), a consultant clinical psychologist working in oncology at St. James University Hospital. Training included teaching about ACT and practice of intervention‐specific therapy methods. This course consisted of two full days of training along with set tasks to complete at home, equating to approximately 5 more hours of home practice. Each site's psychologists had a varied background that may or may not have included previous ACT training prior to our delivered training programme. However, all session leads were Health and Care Professional Council (HCPC) registered practitioner psychologists (Clinical, Health or Counselling Psychologist) who worked with breast cancer patients in a hospital setting
6	How: mechanisms of delivery	The individual session was delivered remotely via video platform by the psychologist. The group sessions (3 in total) were delivered in a group of 8–10 participants, remotely via video platform, also by a psychologist (and a supporting member of staff). Home practice tasks were delivered via the paper participant manual, but they could also be accessed via the bespoke website. Participants were directed to check out the additional information (on side effect management, and extra ACT resources) on the website, which contained written information, audio clips and video clips
7	Where: location of delivery	All sessions were delivered remotely via an on line video platform by a central delivery team at Leeds Hospitals NHS Trust
8	When and how much	The individual session was planned to last for 60 min. Within 6 weeks of this session, the first group session was planned to take place. All three group sessions were planned to last 90 min and were to be held every 2 weeks
9	Tailoring	Although there was a set session plan to follow, which detailed specific exercises and tasks for each session, the therapy itself could be flexible. As such, the deliverer could adapt the content to ensure it was relevant to each participant (e.g., through discussing specific individuals' values, goals and behaviours)
10	Modifications	No modifications were made to the intervention prior to or during intervention delivery
11	How well (planned)	Clinician fidelity to competently deliver the intervention in line with ACT was assessed by an external rater with a background in ACT. They completed the ACT‐FM checklist while listening to the audio recording of 10 sessions. A score of above 28 (out of 72) is considered competent Additionally, an intervention‐specific metric of ‘Procedural Fidelity’ was included, which measured other aspects of the intervention that are important for treatment fidelity but are not ACT‐specific (e.g., giving worksheets, presenting information etc.). It included 7 items for session 1 (individual session), 10 items for the first group session, 9 items for the second group session and 8 items for the third group session. A percentage score was created for each session by dividing the score achieved by the maximum possible score achievable within that session, and multiplying by 100
12	How well (actual)	*ACT‐FM competency* Two independent assessors reviewed and rated the same 10 therapy sessions (5 individual and 5 group) using the ACT‐FM. All four therapists were represented within the randomly selected 10 sessions. Of these 20 ratings, all scored ≥ 39%, which meets the green progression criteria. *Procedural fidelity* Fifty therapy sessions (38 individual and 12 group) were delivered during the trial and of these, 49 (98.0%) scored ≥ 80% on procedural fidelity checklist meeting the green progression criteria. The average procedural fidelity score was 6.8 out of 7 for the individual sessions, and maximum scores were recorded for group sessions 1–3

## APPENDIX B Patient Interview: Interview Schedule

1. Welcome, introduction, basic information capturing (5 min)

Outline aim of this/interview.

Reminder of the intervention.

2 Process of participating (5 min)

**Q**. How did you find the process of signing up to the study? (**Prompts**)

- The consent procedure
- The information received about the study
- The reminder phone calls
- The questionnaires (post vs. online, time taken, any concerns about specific questionnaires etc.)

3 Experienced acceptability of the intervention (45 min)

**ACT session content and processes (20 min)**

**Q:** What did you think of the initial individual session? (*Overall like/dislike, timing, content, deliverer*).

**Q:** Could anything be done to improve the individual session?

**Q:** Were you able to attend all of the group sessions?

**Q:** Did you experience any challenges or difficulties in attending the group sessions? (*what could be done to remove these/overcome them?*)

**Q:** What did you think of the group sessions? (Overall like/dislike, location, timing, content, number of people in the group, deliverer, anything particularly helpful/unhelpful).

**Q:** How did you feel about hearing the experiences of other women who had been affected by breast cancer?

**Q:** Is there anything about the group sessions that could be improved?

**Q:** What did you think of the online delivery of the ACT sessions?

**Q:** What did you think about the participant manual? (*did you read it, how often etc*.)

**Q:** Was there anything you did not understand that was discussed within any of the sessions?

**Q:** Did you experience any problems with doing the techniques discussed in the ACT sessions?

**Q:** What, if any, techniques from the ACT sessions do you think you will continue to use?

**Website (10 min)**

**Q:** Were you able to register and log on to the website?

- **Q: [If yes]** How often did you access it?
- **Q: [If no]** Was there any particular difficulty that stopped you from accessing the website?

**Q:** What did you think of the website? (*Overall like/dislike*).

- **Q:** Was there any content that you found particularly useful?
- **Q:** What content did you dislike or find particularly unhelpful on the website?

**Q:** Did you watch any of the videos? **[If yes]** did you have any thoughts about them?

**Q:** Did you experience any challenges or difficulties in using the website? (*what could be done to remove these/overcome them?*)

**Q:** How could the website be improved?

**Acceptability of ACTION programmer overall (15 min)**

**Q:** What was your overall experience of the ACTION programme? (*likes/dislikes*).

**Q:** How easy or difficult was it for you to engage with the ACTION programme overall? (*could anything have helped with this?*) (*Burden*).

**Q:** How confident are you that you were able to do everything that was required to participate in the ACTION programme? (e.g., *attending sessions, home practice, reading ppt manual, audio files, website etc*) (*self‐efficacy*).

**Q:** What is your understanding of how the ACTION programme works to support women's overall well‐being and medication taking? (*Intervention coherence*).

**Q:** What impact do you think the ACTION programme is likely to have on women's medication taking/overall well‐being? (*Perceived effectiveness*).

**Q:** How does the ACTION programme fit within your own personal values? (*ethicality*).

**Q:** Did participating in the ACTION programme interfere with any of your other priorities or values? (opportunity costs).

**Q:** Would you recommend the ACTION programme to other women in a similar situation to yourself? (*Why/why not?*)

**Q:** Is there anything that you have not already mentioned that you think could be improved about the ACTION programme?

4 Debrief and thank you (3 min)

## APPENDIX C Clinical Psychologists Interview Schedule

1. Welcome, introduction (2 min)

Outline aim of this interview

Reminder of the intervention

2 Views on training (5 min)

**Q:** Could you describe your experience of the training?

**Q:** Were any aspects of the training useful?

**Q:** What part of the training was least helpful in improving your ACT skills?

**Q:** How could the training be improved?

3 Content of Intervention (15 min)

**Q**. What were your overall impressions of the intervention content?

- **Q:** Was there anything you particularly liked about the intervention content (content of the sessions)?
- **Q:** Was there anything you particularly disliked about the intervention content (content of the sessions)?
- **Q:** How could the intervention content be improved?

**Q:** What parts of the intervention do you think that the patients found most useful or responded most positively to?

**Q:** Were there any aspects of the intervention that you found the patients responded less positively or negatively to?

**Q:** What part of the intervention do you think would be most effective/least effective at supporting medication decisions/quality of life?

**Q:** How likely do you think the ACTION programme overall will be in supporting medication decisions/quality of life?

**Q:** What do you think could make the ACTION programme more effective at improving medication decisions/quality of life?

4 Acceptability of format and processes (15 min)

**Q**. What are your overall views regarding the format of the intervention?

**Q:** Was there anything with regard to the format/configuration of the intervention that you liked/worked out well?

**Q:** How could the format/configuration be improved?

**Q:** How easy or difficult was it for you to deliver the intervention?

**Q:** Did you experience any difficulties when delivering this intervention? (*What could be done to remove these/overcome them?*)

**Q:** What is your understanding of how the intervention works to support overall wellbeing and medication taking in these women?

**Q:** How confident do you feel in your ability to deliver the ACT sessions? (*anything that could help with this?*)

**Q:** To what extent does the intervention fit within your values as a therapist?

**Q:** Were there any other priorities that you had to give up in order to deliver this intervention?

5 Improvements (5 min)

**Q:** Do you have any other suggestions of how this intervention, on the whole, be improved? (*Delivery, quality, efficacy?*)

**Implementation** (5 min)

**Q:** Do you think that this intervention, as it is now, could be adapted into routine NHS care for women who have been prescribed hormone therapies? (*If no, why not? What would need to be changed?*)

6 Debrief and thank you (3 min)

## APPENDIX D ACTION Participant RAP Sheet

**Table:**

**Processes**	
Process of participating (*consent, information received, reminder phone calls, questionnaires*)	
**ACT sessions**	
Individual Session content—likes	
Individual Session content—dislikes	
Individual session—other factors (*timing, deliverer*, etc.)	
Individual session—improvements	
Group sessions content—likes/helpful aspects	
Group sessions content—dislikes/unhelpful aspects	
Group sessions—other factors (*attendance, challenges of attendance, location, timing, number of people in group, deliverer*)	
Feelings about hearing experiences of other women affected by breast cancer	
Group session improvements	
Views on online format	
View on materials (*participant manual, audio files*)	
Difficulty understanding and using ACT techniques	
Skills that will continue to be used in the future	
**Website**	
Registering and logging on (*barriers, frequency of access*)	
Content of website—likes/helpful aspects	
Content of website—dislikes/unhelpful aspects	
Thoughts about website videos	
Challenges to using website	
Website Improvements	
**Acceptability of ACTION programme**	
Affective attitude—Overall experience—likes	
Affective attitude—Overall experience—dislikes	
Burden—Ease of engaging with ACTION programme	
Self‐efficacy—confidence in engaging in the ACTION programme	
Perceived effectiveness of ACTION programme	
Intervention coherence—understanding of how ACTION programme will support wellbeing and medication taking?	
Ethicality—how does ACTION fit with personal values?	
Opportunity costs—interference of ACTION with other priorities and values?	
Would you recommend the ACTION programme to other women in a similar situation? (*Why/why not?*)	
ACTION programme improvements	
**Other**	
Miscellaneous	

## APPENDIX E ACTION Therapist RAP Sheet

**Table:**

**Researcher**	
Views on training (*experiences, useful and unhelpful aspects*)	
Improvements to training	
**Content of intervention**	
Views of intervention content—*likes*	
Views of intervention content—*dislikes*	
Improvements to intervention content	
Most helpful aspects of intervention	
Least helpful aspects of the intervention	
Perceived effectiveness	
Improvements to aid effectiveness	
**Acceptability of format and processes**	
Overall views on the format of the intervention—*likes*	
Overall views on the format of the intervention—*dislikes*	
Improvements to format of the intervention	
Burden‐ Ease of delivery of the intervention (*barriers to delivery, what could be done to overcome them*)	
Intervention coherence—understanding of how it will support overall wellbeing and medication taking	
Self‐efficacy in delivering the intervention	
Ethicality—how does the intervention fit with values as a therapist	
Opportunity costs—did you give up anything else to deliver this intervention?	
**Improvements**	
Any additional improvements	
**Implementation**	
Views on implementation	
Changes to allow implementation	
**Other**	
Miscellaneous	
